# Flavocoxid Inhibits Phospholipase A2, Peroxidase Moieties of the Cyclooxygenases (COX), and 5-Lipoxygenase, Modifies COX-2 Gene Expression, and Acts as an Antioxidant

**DOI:** 10.1155/2011/385780

**Published:** 2011-06-19

**Authors:** Bruce P. Burnett, Alessandra Bitto, Domenica Altavilla, Francesco Squadrito, Robert M. Levy, Lakshmi Pillai

**Affiliations:** ^1^Primus Pharmaceuticals, Inc., 4725 North Scottsdale Road, Scottsdale, AZ 85251, USA; ^2^Department of Clinical and Experimental Medicine and Pharmacology, University of Messina, Messina, Italy

## Abstract

The multiple mechanisms of action for flavocoxid relating to arachidonic acid (AA) formation and metabolism were studied *in vitro*. Flavocoxid titrated into rat peritoneal macrophage cultures inhibited cellular phospholipase A2 (PLA_2_) (IC_50_ = 60 *μ*g/mL). In *in vitro* enzyme assays, flavocoxid showed little anti-cyclooxygenase (CO) activity on COX-1/-2 enzymes, but inhibited the COX-1 (IC_50_ = 12.3) and COX-2 (IC_50_ = 11.3 *μ*g/mL) peroxidase (PO) moieties as well as 5-lipoxygenase (5-LOX) (IC_50_ = 110 *μ*g/mL). No detectable 5-LOX inhibition was found for multiple traditional and COX-2 selective NSAIDs. Flavocoxid also exhibited strong and varied antioxidant capacities *in vitro* and decreased nitrite levels (IC_50_ = 38 *μ*g/mL) in rat peritoneal macrophages. Finally, in contrast to celecoxib and ibuprofen, which upregulated the *cox*-2 gene, flavocoxid strongly decreased expression. This work suggests that clinically favourable effects of flavocoxid for management of osteoarthritis (OA) are achieved by simultaneous modification of multiple molecular pathways relating to AA metabolism, oxidative induction of inflammation, and neutralization of reactive oxygen species (ROS).

## 1. Introduction

Osteoarthritis is a multifactorial disease, often caused by injury or repetitive trauma, and involves metabolic as well as inflammatory components. Constant “wear-and-tear” on joints leads to the release of phospholipids from damaged cells which are then converted by PLA_2_ into AA [1]. Dietary habits, particularly excess consumption of AA in the form of omega-6 fatty acids, have been shown to impact the extent and progression of OA in humans [2]. Through the action of the COX and 5-LOX enzymes, AA from tissue destruction and diet is converted into inflammatory metabolites such as thromboxane (TX), prostaglandins (PGs), prostacyclins (PCs), and leukotrienes (LTs) [3, 4]. 

**Figure 1:**
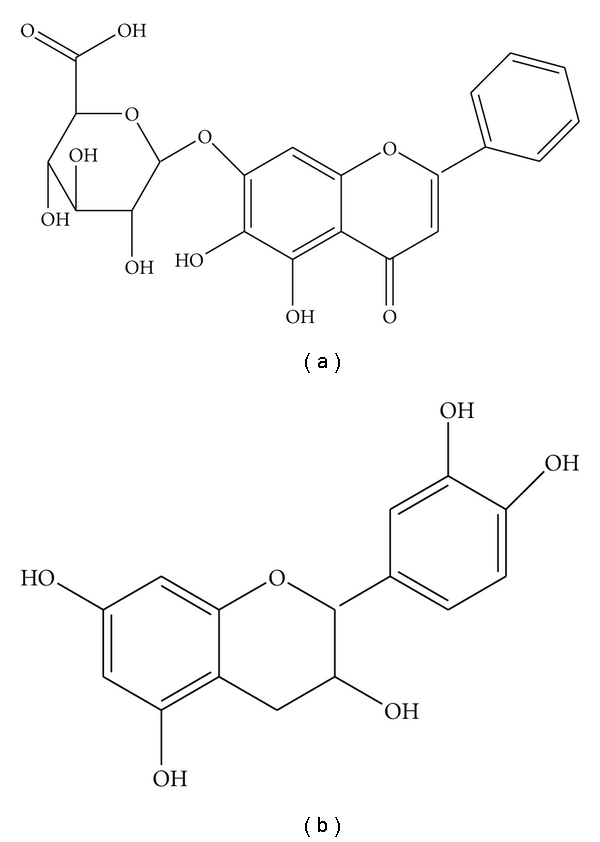
Flavocoxid (a) baicalin and (b) catechin.

Flavocoxid, marketed as Limbrel, is a USFDA-regulated prescription medical food (CFR Volume 21 USC [Code] section 360ee (b)(3)) for the clinical dietary management of the metabolic processes of OA. The molecules in flavocoxid were isolated by high-throughput screening of thousands of natural extracts having anti-PO, COX-1 and COX-2 activity as well as 5-LOX enzyme inhibition [5]. The product is composed of a proprietary mixture of the flavonoid molecules baicalin, extracted from *Scutellaria baicalensis*, and catechin, from *Acacia catechu*, concentrated to greater than 90% purity (Figure 1) [6]. 

Recent clinical efficacy trials utilizing *Garcinia kola *[7], pine bark extract-derived bioflavonoids [8, 9], and flavocoxid itself [10–12] demonstrate a renewed interest in the use of natural molecules for the management of OA. The exact mechanism of action of flavonoid-based therapeutics is unknown. Using a variety of *in vitro *enzyme, cell, gene expression, and antioxidant assays, we examined the anti-inflammatory mechanism of action of flavocoxid. Flavocoxid's inhibitory activity on PLA_2_ as well as its exact interaction with the COX and 5-LOX enzymes was clarified. In addition, the broad antioxidant capacity of flavocoxid and its effect on nitrite production in a cell model was determined and related to its damping of *cox-2* gene expression compared to other anti-inflammatory agents. These pleiotropic, anti-inflammatory effects of flavocoxid are discussed, correlating this *in vitro* characterization to *in vivo* safety and efficacy results in humans for the clinical management of OA. 

## 2. Materials and Methods

### 2.1. Materials

The following NSAIDs were purchased from Sigma-Aldrich (St. Louis, Mo, USA): meloxicam, sodium salt (#3935); naproxen, sodium salt (#5160); diclofenac, sodium salt (#D6899); ibuprofen, sodium salt (#I1892) and NS-398 (#N194). Rofecoxib was purchased from Merck & Co. Inc. (Whitehouse Station, NJ, USA), and valdecoxib and celecoxib from Pfizer (New York, NY, USA). Flavocoxid, as well as 90% pure baicalin and 90% pure catechin, were provided by Primus Pharmaceuticals, Inc. (Scottsdale, Ariz, USA). Each compound was dissolved in 100% dimethyl sulfoxide (DMSO; Sigma-Aldrich). Flavocoxid is a mixture of compounds with different molecular weights. Therefore, all concentrations are given in *μ*g/ml for the compounds tested rather than micromolar concentrations. 

### 2.2. Methods

#### 2.2.1. Animals and Isolation of Peritoneal Macrophages for Nitrite Determination and Phospholipase A2 Assay

All procedures complied with the standards for care and use of animal subjects as stated in the Guide for the Care and Use of Laboratory Animals (Institute of Laboratory Animal Resources, National Academy of Sciences, Bethesda, Md, USA). Peritoneal macrophages were obtained from male Sprague–Dawley rats (250–275 g) by washing the abdominal cavity with RPMI 1640. The cells were centrifuged twice and resuspended in the same medium at a concentration of 3 × 10^6^/mL Macrophages were purified after 2 h adhesion to plastic Petri dishes (Nunc, Roskilde, Denmark) at 37°C. The homogeneity and the viability of macrophages were greater than 98% as determined by differential staining and trypan blue exclusion. Macrophages were stimulated for 6 h with 1 *μ*g/mL of lipopolysaccharide (LPS). Lipopolysaccharide-stimulated macrophages were coincubated with flavocoxid at 10, 20, 50, 100, 200, and 500 g/mL or RPMI medium alone.

**Cell Viability**

Cell viability of peritoneal macrophages, following exposure to flavocoxid (from 10, 20, 50, 100, 200 and 500 *μ*g/mL) and/or with 1 *μ*g/mL LPS at 37°C, was determined after 24 h of incubation by the MTT [3-(4,5-dimethylthiazol-2-yl)-2,5-diphenyltetrazolium bromide] assay, as described by Mosmann [13].

**Phospholipase A2 Assay**

Phospholipase A2 was assayed in cytosolic extract by using the cPLA_2_ Assay kit (Cayman, Ann Arbor, Mich, USA) following the manufacturer's protocol. Briefly cells were homogenized in lysis buffer (50 mM Hepes pH 7.4, 1 mM EDTA), sonicated and then centrifuged at 10.000 × g for 15 min at 4°C. The supernatant was removed for the assay and stored on ice. Absorbance was read at OD4_14__nm_, and results were compared to the positive control (bee venom PLA_2_).

#### 2.2.2. Purified Enzyme Assays to Test Flavocoxid's Effect on COX-1 and COX-2 Cyclooxygenase and Peroxidase Activities and 5-LOX Inhibition

**COX-1 and COX-2 Cyclooxygenase Inhibition Assay**

Flavocoxid was dissolved in 100% DMSO at a stock concentration of 10 mg/mL. Flavocoxid was tested in triplicate at 0, 0.1, 1, 10 and 50 *μ*g/mL. DMSO alone alters oxygenase activity. Therefore, the highest concentration of flavocoxid that could be tested using the oxygraph assay (Cayman Chemical Company, Inc.) to determine CO-specific inhbition without interference was 50 *μ*g/mL. Indomethacin (MW = 357.8) and NS-398 (MW = 314.4) were run as positive controls for inhibition of COX-1 and COX-2, respectively. Indomethacin was tested in triplicate at 0, 3.6, 18, 36, 180, and 360 ng/mL. NS-398 was tested in triplicate at 0, 3.1, 15.7, 31.4, 157 and 314 ng/mL. These inhibitors were pre-incubated with the enzyme in reaction buffer for one minute prior to the addition of AA. Assays were performed using 100 units of ovine COX-1 or ovine COX-2 (one unit of enzyme consumes one nanomole of oxygen per minute at 37°C in 0.1 M Tris-HCl buffer, pH 8.0, containing 20 *μ*M AA, 5 mM EDTA, 2 mM phenol, and 1 *μ*M hematin). Assays were initiated by the addition of 20 *μ*M AA, and oxygen consumption measured using a Gilson Model 5/6H oxygraph equipped with a Clark oxygen electrode [14].

**COX-1 and COX-2 Peroxidase Inhibition Assays**

A cleavable, peroxide chromophore (N,N,N′N′-Tetramethyl-p-phenylenediamine dihydrochloride (TMPD)) (Sigma) was included in the assay to measure the inhibitor effect on PO activity of COX-1 and COX-2 in the presence of AA as a cofactor [15]. Each inhibitor was tested for PO-specific inhibition in triplicate (Cambridge Biomedical Research Group, Inc.), at room temperature (25°C) over a range of concentrations from 0 to 500 *μ*g/mL using ovine COX-1 and COX-2 enzymes (provided by Cayman Chemical, Ann Arbor, Mich, USA), hematin (Sigma), TMPD (Sigma), and AA (Sigma). Absorbance of the cleaved TMPD substrate was read at OD_570 nm_. The mean results for each inhibitor concentration versus percentage inhibition was plotted, and the IC_50_ determined by taking the half-maximal point along the isotherm and intersecting the concentration on the *x*-axis.

**5-LOX Inhibition Assay**

A Lipoxygenase Inhibitor Assay Kit (Cayman Chemical) was used in which potato 5-LOX (Cayman Chemical) was substituted for the soy 15-LOX usually present in the kit. The buffer was also changed from phosphate buffer, pH 6.8 to a Tris-HCl, pH 7.4 buffer and was used to measure 5-LOX inhibition for each inhibitor. This assay detects the formation of unstable hyperoxides, HPETEs, which are then converted to LTs through an oxygen sensing chromagen system consisting of FeSO_4_·7H_2_O and NH_4_SCN that changes to bright yellow [16]. The assay was performed in triplicate using potato 5-LOX (15.3 units), linoleic acid (LA) as a cofactor, and TMPD as a substrate with a variety of inhibitors titrated from 0 to 500 *μ*g/mL and flavocoxid from 0 to 1000 *μ*g/mL at room temperature. Absorbance of the cleaved TMPD substrate was read at OD_500 nm_. The mean results for each inhibitor concentration versus percentage inhibition was plotted and the IC_50_ determined by taking the half-maximal point along the isotherm and intersecting the concentration on the *x*-axis.

#### 2.2.3. In Vitro Antioxidant Capacity Assays of Flavocoxid

**Nitrite Production**

Nitrite levels, a breakdown product of nitric oxide (NO), were measured in a standard Griess reaction. Briefly, 100 *μ*L of LPS-stimulated macrophage supernatants with and without flavocoxid were incubated with an equal volume of Griess reagent (1% sulphanilamide and 0.1% naphthyl-ethylenediamine dihydrochloride in 2.5% phosphoric acid). After 10 min of incubation at room temperature, the absorbance of the chromophore was measured at OD_540 nm_ using a microtitre plate reader. Nitrite concentrations were calculated by comparison with a standard calibration curve with sodium nitrite (NaNO_2_: 1.26 to 100 mM), with control baseline supernatant as the blank.

**Antioxidant Assays**

The *in vitro* antioxidant activity of flavocoxid was evaluated using Oxygen Radical Absorbance Capacity (ORAC) procedures (Brunswick Laboratories, Norton, Mass, USA). Values are expressed as *μ*mol of Trolox equivalents (TE) per g of dry weight and compared to known values for vitamins C and E. The ORAC analysis provides a measure of the scavenging capacity of antioxidants against the peroxyl radical, which is one of the most common ROS found in the body. ORAC_hydro_ reflects water-soluble antioxidant capacity, and the ORAC_lipo_ is the lipid soluble antioxidant capacity. Trolox, a water-soluble Vitamin E analog, is used as the calibration standard, and the ORAC result is expressed as *μ*mol TE/g dry weight.

The ferric reducing/antioxidant power (FRAP) assay was performed as previously described [17]. A validated assay for hydroxyl radical absorbance capacity (HORAC) was also performed according to described methods [18]. The peroxynitrite radical averting capacity assay (NORAC) and superoxide radical averting capacity (SORAC) assays were performed as described previously [19].

Other antioxidant capacity assays used in the analysis of flavocoxid's antioxidant capacity include TEAC (trolox equivalent antioxidant capacity), a method developed by Rice-Evans' group and broadly applied in analyzing food samples [20] and DPPH [2,2-di(4-*tert*-octylphenyl)-1-picrylhydroxyl], an easy and accurate method frequently used to measure the antioxidant capacity of fruit and vegetable juices and extracts [21].

#### 2.2.4. Effects of Flavocoxid, Celecoxib, Ibuprofen, and Acetominophen on cox-1 and cox-2 Gene Expression

Human peripheral blood mononuclear cells (PBMCs) were isolated from apheresis products (COBE Laboratories, Inc.) using a Histopaque density gradient [22] and cocultured at ~4.5 × 10^6^ cells in 3 mL growth medium per well in 6-well plates with LPS at 10 ng/mL at 37°C in a humid environment with 5% CO_2_. The samples were collected 18 hr posttreatment. Total RNAs were prepared using the Qiagen RNeasy Kit and cDNAs synthesized using the ABI cDNA Archive kit, following the protocols of the suppliers. QPCR assays were run in duplicate in an ABI 7700 Sequence Detector using ABI TaqMan Gene Expression primer and probe sets for COX-1 and COX-2. Flavocoxid was compared to celecoxib, ibuprofen, and acetaminophen all at 3 *μ*g/mL for their effects on *cox-1* and *cox-2* expression. Cyclophilin A was used as the reference transcript for the relative quantification of RNA levels to normalize gene expression.

### 2.3. Statistical Analysis

All data are expressed as the mean ± SD. Data were assessed by analysis of variance for multiple comparisons of results. The Duncan multiple range test was used to compare group means. In all cases, a probability error of less than  .05 was selected as the criterion for statistical significance.

## 3. Results

**PLA_2_ Inhibitory Activity**

Other well researched flavonoids, such as green tea catechins and quercitin have been shown to inhibit PLA_2_ thus modulating the generation of AA from membrane phospholipids [23, 24]. Little is known, however, regarding the direct inhibitory effects of either baicalin or catechin on PLA_2_ activity. Therefore, flavocoxid was tested in a macrophage cell assay for its ability to inhibit PLA_2_ activity.

**Figure 2:**
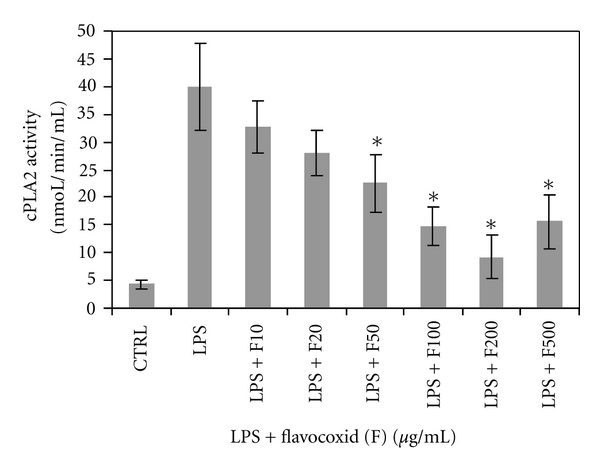
Cellular phospholipase A2 activity in rat peritoneal macrophages exposed to flavocoxid (F) at 10, 20, 50, 100, 200, and 500 *μ*g/mL in the presence of lipopolysaccharide (LPS).

Flavocoxid had a minor, nonstatistical effect on macrophage cell viability at 200 and 500 *μ*g/mL (data not shown). Phospholipase A2 inhibition levels were corrected for cell viability. When flavocoxid was titrated into LPS-stimulated rat peritoneal macrophage cultures, it exhibited a dose response inhibition of PLA_2_ (IC_50_ = 60 *μ*g/mL) at concentrations of 50, 100, 200, and 500 *μ*g/mL significantly better than LPS alone (*P* < .05) (Figure 2). This result suggests that flavocoxid has the ability to modulate the generation of AA from membrane phospholipids produced by the destruction of tissue which occurs in OA.

**COX-1 and COX-2 Cyclooxygenase Inhibitory Activity**

The COX proteins contain two different enzymatic moieties for metabolism of AA, cyclooxygenase (CO) and peroxidase (PO). The CO activity converts AA to PGG_2_ and the PO activity metabolizes PGG_2_ to PGH_2_ [25]. Subsequent platelet and cellular synthases as well as isomerases then convert PGH_2_ to TX as well as PGs and PCs. Medications used to treat OA, such as traditional NSAIDs and selective COX-2 inhibitors, block the production of PGG_2_, but do not affect the PO site. Flavocoxid was tested for its specific CO and PO inhibition using purified enzymes *in vitro* to define its specific anti-COX-1 and COX-2 effects.

**Figure 3:**
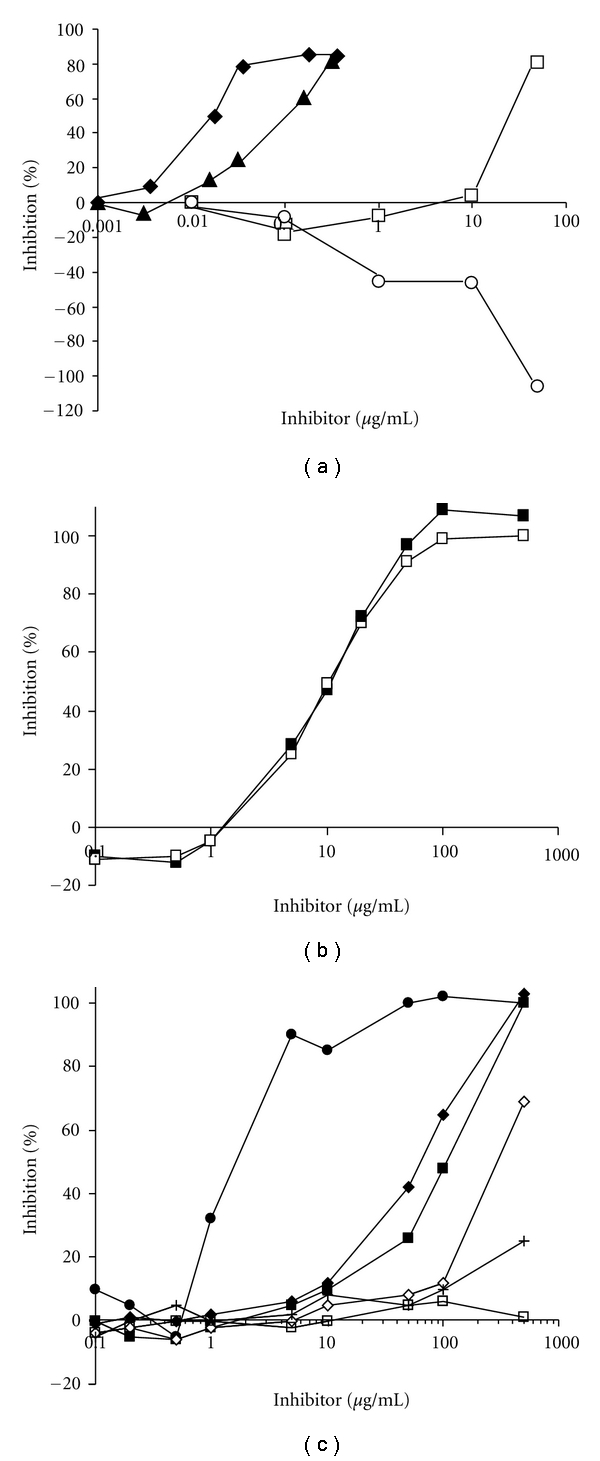
(a) Flavocoxid (0, 0.1, 1, 10, 50 *μ*g/mL) COX-1 (□) and COX-2 (○) cyclooxygenase (CO) inhibition versus indomethacin (0, 3.6, 18, 36, 180, 360 ng/mL, ♦) and NS-398 (0, 3.1, 15.7, 31.4, 157, 314 ng/mL, ▲). (b) Flavocoxid (0.1, 0.5, 1, 2, 5, 10, 50, 100, 500 *μ*g/mL) COX-1 (■) and COX-2 (□) peroxidase (PO) inhibition. (c) Inhibition of 5-Lipoxygenase for phenidone (●), baicalin (♦), flavocoxid (■), catechin (*⋄*), ibuprofen (+), and celecoxib (□) at 0.1, 0.2, 0.5, 1, 5, 10, 50, 100, 500 *μ*g/mL.

Flavocoxid showed no detectable anti-CO COX-2 activity up to 50 *μ*g/mL demonstrated by a downturn in the curve and accumulation of oxygen in the assay (Figure 3(a)). NS-398, a strong selective COX-2 inhibitor, had a CO COX-2 IC_50_ of 0.095 *μ*g/mL. Compared with indomethacin, which gave a CO COX-1 IC_50_ of 0.012 *μ*g/mL, flavocoxid had an IC_50_ of 25 *μ*g/mL. Therefore, indomethacin was more than 2000-fold stronger in inhibiting CO COX-1 than flavocoxid. These results suggest that flavocoxid has little anti-CO activity on the COX enzymes compared to well-characterized anti-inflammatory agents.

**COX-1 and COX-2 Peroxidase Inhibitory Activity**

It has been shown that imbalances in COX-2 versus COX-1 inhibition by selective COX-2 inhibitors in the generation of various AA metabolites can contribute to edema, hypertension, and myocardial infarctions [26]. Thus, the specially formulated, proprietary mixture of baicalin and catechin has been designed in an attempt to balance COX-1 and COX-2 metabolism of AA focusing on inhibition of the PO activity of these enzymes. A cleavable, peroxide chromophore TMPD was used to assess the anti-PO activity of flavocoxid on purified enzyme systems using COX-1 and COX-2.

Analysis of 90% pure baicalin used to formulate flavocoxid was slightly more selective against the COX-2 (IC_50_  = 10 *μ*g/mL) compared to COX-1 PO activity (IC_50_  = 13 *μ*g/mL) activity (data not shown). The 90% pure catechin used in the formulation, however, showed preferential inhibitory activity towards the PO of COX-1 (IC_50_  = 2.5 *μ*g/mL) versus COX-2 (IC_50_  = 15 *μ*g/mL) (data not shown). In an attempt to balance the PO COX-1 with that of PO COX-2 inhibition activity, baicalin and catechin was combined at a proprietary ratio to form flavocoxid. Flavocoxid showed relatively balanced inhibition of both COX-1 and COX-2 PO activities with IC_50_s of 12.3 and 11.3 *μ*g/mL, respectively (Figure 3(b)). These results as well as the relative lack of CO activity on COX-1 and COX-2 suggest that flavocoxid exerts its effects via modulation of the PO activity of these enzymes.

**5-LOX Inhibitory Activity**

NSAIDs and COX-2 inhibitors do not inhibit the 5-LOX pathway to prevent the accumulation of leukoattractive and vasoconstrictive LTs [27]. Inhibition of COX-1 and COX-2 by NSAIDs or selective COX-2 inhibitors has also been shown to “shunt” AA metabolism down the 5-LOX pathway, resulting in an overabundance of these fatty acid metabolites which can adversely affect multiple organs [28]. Increased levels of LTs are associated with a variety of pathological conditions including asthma, gastric ulcerations, renal insufficiency, and cardiovascular complications [29, 30]. Cyclooxygenase enzyme inhibition has also been shown to increase the level of LTB_4_ in synovial fluid, perhaps inducing further damage to cartilage [31]. Flavocoxid was titrated along with purified 5-LOX enzyme in the presence of an oxygen sensing chromogen *in vitro* to detect the formation of unstable HPETEs, an intermediate in the formation of LTs.

Individual tests of baicalin and catechin revealed differences in 5-LOX inhibitory capacity. Baicalin exhibited a relatively strong inhibition of the 5-LOX enzyme with an IC_50_ of 65 *μ*g/mL in comparison to catechin (IC_50_  = 300 *μ*g/mL) (Figure 3(c)). Flavocoxid, presumably reflecting the ratio of these two molecules in the formulation, inhibited the 5-LOX enzyme with an IC_50_ of 110 *μ*g/mL. With the exception of the known 5-LOX inhibitor, phenidone (IC_50_  = 1.3 *μ*g/mL), used as a positive control in these assays, no other NSAID or selective COX-2 inhibitors, including rofecoxib, valdecoxib, diclofenac, meloxicam, and aspirin (data not shown), showed any anti-5-LOX activity. Ibuprofen with a small amount of 5-LOX inhibition at the highest concentration tested (no IC_50_ could be determined) is also shown in comparison to celecoxib (Figure 3(c)). These results suggest that flavocoxid also modulates the formation of LTs from 5-LOX and may prevent an accumulation of these key inflammatory factors which contribute to tissue damage through a putative 5-LOX shunt seen with NSAIDs.

**Antioxidant Capacity**

Nonenzymatic lipid peroxidation is another important pathway of AA metabolism. When elevated AA is present in organisms and it is exposed to ROS, F2-isoprostanes and 4-hydroxynonenal (HNE) are produced along with elevated malondialdehyde (MDA) in the presence of epinephrine and collagen [32]. These oxidative conversion products are elevated in the synovial fluid, synoviocytes, and serum of OA patients when compared to healthy control subjects and have been shown to stimulate the production of cartilage-degrading matrix metalloproteinases [33]. No NSAIDs used in the treatment of OA possess potent antioxidant properties which could potentially damp inducible inflammation [27]. Flavocoxid modulates the production of inflammatory cytokines and inducible nitrous oxide synthase (iNOS) in cell assays presumably through an antioxidant mechanism of action [5, 34], but the extent of its antioxidant capacity has never been investigated. To evaluate the antioxidant capacity of flavocoxid, a number of *in vitro* antioxidant assays were used.

The ORAC_total_ for flavocoxid was found to be higher than the control antioxidants vitamin C (ORAC_total_  = 2000 *μ*molTE/g) and vitamin E (ORAC_total_  = 1100 *μ*molTE/g) at 3719 *μ*molTE/g with the ORAC_lipo_ (19 *μ*molTE/g) contributing little to the overall ORAC score (Table 1). Flavocoxid had a HORAC value of 1326 *μ*mol CAE/g for hydroxyl radicals, which have also been implicated in joint damage. Similarly, peroxynitrite may cause chondrocytes to decrease synthesis of cartilage proteoglycan. Flavocoxid, with a NORAC value of 1936 *μ*molTE/g, has a high capacity to neutralize peroxynitrite radicals. On the contrary, superoxide radical scavenging capacity (SORAC) is relatively low for flavocoxid (Table 1). Shieh et al. [35] found that baicalein had a much higher superoxide anion capacity compared to baicalin. This may explain the relatively low SORAC value of flavocoxid, as baicalin, rather than baicalein, the gut bacterial breakdown product absorbed systemically from flavocoxid, was tested in this assay. With respect to DPPH, our results are consistent with others that show baicalin and epicatechin, the stereoisomer of catechin, to be strong DPPH scavengers [36]. Flavocoxid also showed a high FRAP value. Our results also show that flavocoxid has a high TEAC of 2456 *μ*molTE/g (Table 1).

**Nitrite Suppression by Flavocoxid**

Stable nitrite is used as a measure of NO produced from iNOS from synovial macrophages in OA [37]. Nitric oxide may be involved in the destruction of proteoglycan in cartilage by inducing the production of matrix metalloproteinase [38]. To assess flavocoxid's effect on the production of this highly reactive oxidative molecule, LPS-stimulated rat peritoneal macrophages expressing higher levels of iNOS were used in the presence of increasing levels of the flavonoid preparation. Nitrite measured by Griess reaction and subsequent spectrophotometric analysis measured flavocoxid's NO damping capacity.

**Figure 4:**
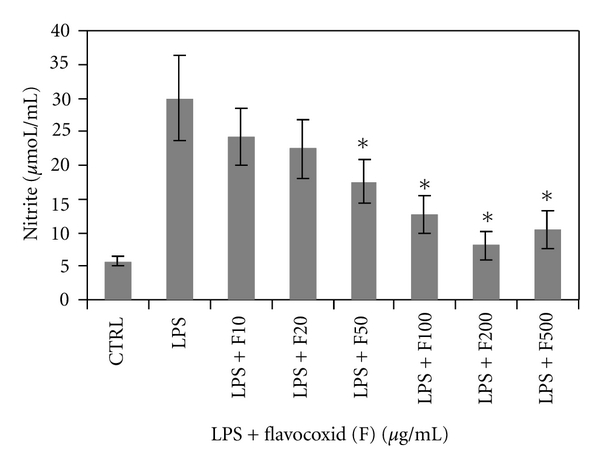
Nitrite levels in rat peritoneal macrophages exposed to flavocoxid (F) at 10, 20, 50, 100, 200, and 500 *μ*g/mL in the presence of lipopolysaccharide (LPS).

When flavocoxid was titrated into cultures, it suppressed nitrite production in a dose-dependent manner with an IC_50_ of 38 *μ*g/mL compared to cultures with LPS alone (*P* < .05) (Figure 4), suggesting that flavocoxid downregulates the production of or neutralizes NO directly. Flavocoxid's activity in reducing NO production through damping of iNOS [5] or through inactivation of NO by a direct antioxidant effect could help prevent to breakdown of the proteoglycan in cartilage.

**Flavocoxid Effects on Cox-1 and Cox-2 Gene Expression**

Inducible inflammatory genes are generally up-regulated by the generation of ROS which induce *nf*κ*b* expression and activation by release of cytosolic I**κ**B*α*-bound NF**κ**B from the cytoplasm and subsequent translocation to the nucleus [27]. Cytokine (*il-1*β**, *il-6* and *tnf*α**) as well as *cox-2*, *5-lox* and *inos* genes have an NF**κ**B binding site in their promoter regions. *Cyclooxygenase-1* gene expression is not inducible, but is regulated through intron elements to yield constitutively produced protein in many cell types. Flavocoxid is known to damp inducible inflammatory gene and protein production [5], but has not been compared directly to other NSAIDs or analgesics for this activity. Therefore, flavocoxid was added to PBMCs at a fixed concentration and compared directly to celecoxib, ibuprofen, and acetaminophen for its effects on *cox-1* and -*2* expression.

**Figure 5:**
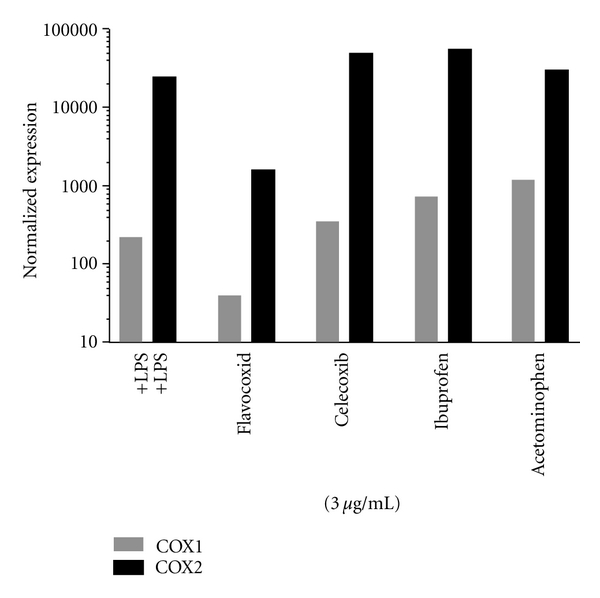
Effect of flavocoxid versus celecoxib, ibuprofen, and acetominophen on *cox-1* (gray) and *cox-2* (black) gene expression.

When flavocoxid was added at 3 *μ*g/mL into cultures of PBMCs stimulated with LPS, there was a 20-fold down-regulation of *cox-2* (Figure 5), but only a 3-fold reduction in *cox*-1 expression. At the same concentration, celecoxib, ibuprofen, and acetominphen did not reduce *cox* expression. In fact, celecoxib and ibuprofen increased *cox*-2 expression 2 to 3 fold with acetaminophen exhibiting less than a doubling of gene expression. Celecoxib, ibuprofen, and acetaminophen also increased *cox-1* expression 1.2-, 5-, and 8-fold, respectively (Figure 5). These results suggest that flavocoxid may modulate inflammatory metabolites, such as PG, by reducing *cox* gene expression compared to celecoxib, ibuprofen and acetaminophen which cause increased levels of *cox-1 *and* cox-2* expression.

## 4. Discussion

An overabundance of AA in the general population's diet of Western countries has shifted the balance of fatty acid metabolism toward an increase in proinflammatory metabolite generation via the COX and 5-LOX enzymatic pathways [39, 40]. This has been accompanied by an increased generation of lipid peroxidation products by AA-oxidation as a result of poor antioxidant status. There is a strong link between metabolic defects in fatty acid metabolism or an overabundance of dietary fatty acids and the incidence of OA. For example, fatty acid levels in bone have been shown to be 50–90% higher in OA patients compared to controls [41]. Poor oxidative status, as well as the production of oxidized lipids from AA, has also been linked to chondrocyte apoptosis, activation of latent matrix metalloproteinases, and cartilage matrix degradation due to upregulation of inflammatory gene expression [38, 42]. Therefore, an anti-inflammatory agent with pleiotropic activities could help modulate these predisposing dietary factors. Kim et al. [43] reviewed this subject and found that a wide variety of flavonoids modulate the activities of AA metabolizing enzymes, such as PLA_2_, COX, and 5-LOX and the NO producing enzyme, iNOS. We sought to test flavocoxid, a prescription product, to determine its impact on these pathways which contribute to OA.

Lipid peroxidation, as a result of poor oxidative status, destabilizes cell membranes leading to induction of calcium-dependent PLA_2_ which hydrolytically attacks phospholipids leading to the generation of AA [44]. In joints, breakdown of chondrocyte cell membranes provides the substrate for PLA_2_ conversion to AA [45]. With their potent antioxidant capacity, flavonoids can interrupt the oxidative generation of AA from phospholipids and reduce the downstream production of inflammatory metabolites from AA metabolism, oxidative damage, and induction of inducible inflammatory pathways [34, 46–49]. In our study, flavocoxid reduced both PLA_2_ activity and nitrite levels in macrophages. This suggests that flavocoxid may act to limit the conversion of phospholipids from damaged cell membranes to AA upstream of the COX and 5-LOX metabolic pathways. In addition, the reduction in nitrite, the stable breakdown product of NO, may prevent the production of matrix metalloproteinase from macrophages present in the synovium [37]. In support of these findings, flavocoxid was previously shown to reduce iNOS protein production by an antioxidant mechanism [5]. Since it is also known that NO levels are positively affected by increases in cytokine production [50], flavocoxid's cytokine-reducing effect [5, 48] may inhibit the increase in *inos* expression and NO levels.

The current experiments demonstrate that, unlike NSAIDs, flavocoxid does not appreciably inhibit CO metabolism of AA to PGG_2_. Rather it acts *via* a balanced anti-PO inhibitory on the COX enzymes. There are conflicting reports that the PO and CO activities are separable in the COX enzymes [51, 52]. Individual PO and CO mutants have been created *in vitro* [53]. When the PO site was mutated, there were delays in the metabolite generation implying that some other cellular peroxidase may compensate for the mutated activity. Therefore, CO and PO activities are coupled in the COX-1 and COX-2 enzymes to efficiently produce end products [54]. The inhibition of one or both activities reflects modulation of overall COX enzyme activity [55]. Flavocoxid was shown to downregulate both PGE_2_ and LTB_4_ production in cell and animals models of inflammation [5, 34, 48, 56]. Flavocoxid did not, however, block the production of TxA_2_ in a human platelet function study [57], suggesting the presence of another compensatory mechanism that bypasses its PO inhibitory activity on the COX enzymes to allow for generation of the PGH_2_ intermediate which is then converted to TxA_2_. This differential mechanism of PO versus CO inhibition also explains flavocoxid's lack of interaction with aspirin since aspirin specifically modifies the COX-1 binding site [57].

It has been demonstrated that patients with OA of the knee have higher than normal levels of oxidative species and antioxidant enzymes in their synovial fluid [58]. Pure tea catechins and (+)-catechin, found in flavocoxid, are known to be strong antioxidants with high ORAC scores between 13,000 and 20,000 *μ*mol TE/g, respectively [59, 60]. These catechin molecules also have high FRAP and DPPH values. Baicalin was shown, however, to have a much lower ORAC score of ~365 *μ*molTE/g [61]. Although flavocoxid has a lower antioxidant capacity compared to catechin itself, the ORAC_total_ score is still approximately 3 times that of mixed tocopherols and almost twice that of vitamin C. Altavilla et al. [5] showed that flavocoxid's antioxidant capacity reduced cellular conversion of AA to MDA in the presence of epinephrine and collagen normally elevated in OA patients. Nuclear factor-**κ**B binding to a model substrate and I*κ*B*α* protein expression was also elevated in the same experiments suggesting that flavocoxid restored the cytoplasmic inhibitory control of this ROS-inducible transcription factor. An impure mixture of baicalin and catechin has also been shown to decrease oxidative species in synovial fluid in humans with OA suggesting a direct antioxidant effect on the level of ROS species in the joint [62]. Flavocoxid's strong antioxidant capacity also affects inducible inflammatory gene and protein expression.

Previous experiments have demonstrated that flavocoxid downregulates COX-2 and 5-LOX protein expression, but not COX-1 [5]. Tseng-Crank et al. [63] recently showed that a combination of baicalin and catechin dramatically decreases *cox-2* and cytokine expression, but has only a marginal effect on *cox-1* in a human cell model. We found that flavocoxid also decreases *cox-2* expression by ~20-fold in human PBMCs, while having a much smaller effect on *cox-1* (~3-fold). This work showed, however, that celecoxib and ibuprofen promoted noticeable increases in *cox-1* and *cox-2*, suggesting both therapies could potentially contribute to inducible pathways of inflammation. When combined with a strong antioxidant capacity and previous findings on damping inflammatory gene expression [5, 34, 48, 63], the results presented here suggest that flavocoxid acts through an antioxidant mechanism to control inducible inflammatory gene expression in addition to modulating fatty acid processing enzymes including 5-LOX.

Wallace and Ma [64] suggested that metabolic shunting toward the 5-LOX pathway was a major contributor to gastric injury in humans. When gastric mucosa was stimulated with calcium ionophore or arthritis patients were exposed to NSAIDs, there was a reduction in the release of PGE_2_ and an increase in leukoattractive LTB_4_ and vasoconstrictive LTC_4_ and LTD_4_ [65, 66]. Compatible with these findings, a long-term clinical trial has shown that flavocoxid has significantly fewer upper gastrointestinal adverse events compared to naproxen [12, 67]. Similarly, a phase-IV-like postmarketing study showed that flavocoxid was well-tolerated in previously NSAID (including celecoxib) intolerant patients who ceased or decreased gastroprotective use by over 30% [11]. Walton et al. [68] performed a cost analysis which suggests that this safety advantage would make flavocoxid less expensive compared to generic naproxen in a medicare population over a one-year time frame. A well-controlled endoscopy study is needed to definitively judge flavocoxid's gastrointestinal safety.

 Gambaro [69] has suggested that dual inhibition of COX and 5-LOX enzymes may also offer advantages in terms of renal toxicity, which may also affect CV complications. Both NSAIDs and selective COX-2 inhibitors are known to cause renal damage. Levy et al. [12, 67] found that there were statistically fewer incidences of edema for flavocoxid compared to naproxen. Though not reaching statistical significance, creatinine levels trended lower in the flavocoxid group and higher in the naproxen group. Specific well-controlled studies in renal compromised subjects are needed to truly assess flavocoxid's safety in these patients. 

**Figure 6:**
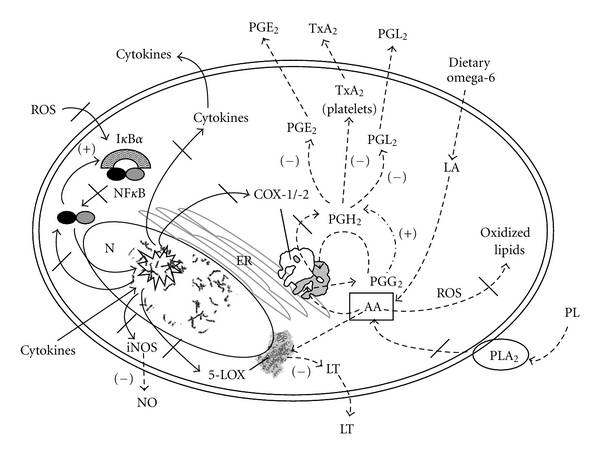
Flavocoxid's putative mechanism of action inhibiting nuclear (N) ROS activation (black slash) of NF*κ*B which induces gene expression (star burst) of *cox-2*,* 5-lox*, cytokines, and *inos* (black slash) through a strong antioxidant stimulatory activity. Dietary omega-6 fatty acids are converted to linoleic acid (LA) (dotted line) and then to AA which are processed through the COX and 5-LOX enzymes. Flavocoxid works on the protein level to limit the production of AA from phospholipids (PL) by inhibiting PLA_2_. It also has been shown to modulate (−) PGE_2_, PGI_2_, and TxA_2_ generation (dotted line) via an anti-PO activity of endoplasmic reticulum (ER)-anchored COX-1 and COX-2 as well as inhibition of nucleus (N)-associated 5-LOX to decreased LTB_4_ production (−). PGH_2_ may be produced (+) from PGG_2_ through and alternate peroxidase activity (dash and dotted line). Flavocoxid has also been shown to restore I*κ*B*α* regulation of NF*κ*B (+).

Flavocoxid is the only currently marketed prescription anti-inflammatory agent that modulates COX enzymes *via* an anti-PO activity, inhibits 5-LOX-mediated LT production, and has a broad, strong antioxidant activity which downregulates inducible inflammatory gene expression as well as neutralizes ROS thereby preventing the conversion of AA to oxidized lipids. Flavocoxid's putative mechanism of action is shown in Figure 6 and is presumed to be the reason for the favorable safety profile seen in clinical trials and postmarketing surveillance. Work is currently underway to determine if this broad-based mechanism of action can preserve cartilage structure. 

## Figures and Tables

**Table 1:** 

	ORAC_hydro_ (*μ*molTE/g)	ORAC_lipo_ (*μ*molTE/g)	ORAC_total_ (*μ*molTE/g)	HORAC (*μ*mol CAE/g)	NORAC (*μ*mol TE/g)	SORAC (kunit SODeq/g)	FRAP (*μ*mol TE/g)	TEAC (*μ*mol TE/g)	DPPH (*μ*mol TE/g)
flavocoxid	3700	19	3719	1326	1936	27	1145	2456	767

ORAC_hydro_: Oxygen Radical Absorbance Capacity reflects water-soluble antioxidant capacity; ORAC_lipo_: Oxygen Radical Absorbance Capacity lipid-soluble antioxidant capacity; ORAC_total_: Combined ORAC_hydro_ and ORAC_lipo_; HORAC: hydroxyl radical absorbance capacity; NORAC: peroxynitrite radical averting capacity; SORAC: superoxide radical averting capacity; FRAP: ferric reducing/antioxidant power; TEAC: trolox equivalent antioxidant capacity; DPPH: 2,2-di(4-*tert*-octylphenyl)-1-picrylhydroxyl assay; TE: trolox equivalents; CAE: caffeic acid equivalents; SODeq: superoxide dismutase equivalents.
